# Silent Invasion of the Rectum: Aggressive Anorectal Melanoma With Pelvic Mass Effect, Venous Compression, and Hepatic Metastases

**DOI:** 10.7759/cureus.106339

**Published:** 2026-04-02

**Authors:** Claudia Pedreira, Daniela Prado Escobar, Kian Memari, Shane Williams, Lissette P Lazo, Peter Cohen

**Affiliations:** 1 Family Medicine, Nova Southeastern University Dr. Kiran C. Patel College of Osteopathic Medicine, Fort Lauderdale, USA; 2 Internal Medicine, Nova Southeastern University Dr. Kiran C. Patel College of Osteopathic Medicine, Clearwater, USA; 3 Family Medicine, Palmetto General Hospital, Hialeah, USA

**Keywords:** anorectal melanoma, metastatic melanoma, mucosal melanoma, obstructive uropathy, pelvic malignancy, rectal melanoma, venous thromboembolism

## Abstract

Anorectal melanoma is a rare and aggressive malignancy arising from melanocytes within the mucosal epithelium of the anorectal region. Because presenting symptoms such as rectal bleeding frequently mimic benign anorectal conditions, diagnosis is often delayed until advanced stages of the disease. Mucosal melanoma is characterized by aggressive local invasion and early metastatic spread.

We present the case of a 54-year-old female with metastatic rectal melanoma whose disease course was complicated by extensive pelvic tumor burden, malignant venous compression leading to deep venous thrombosis, obstructive uropathy, and tumor-associated hemorrhage. Cross-sectional imaging demonstrated a massive posterior pelvic mass occupying the rectouterine space with displacement of surrounding structures, additional rectal tumor extension, metastatic lesions in the liver, and locally invasive disease adjacent to the patient’s colostomy. Diagnosis was confirmed through sequential biopsy, molecular analysis, and definitive surgical pathology, demonstrating a poorly differentiated malignant neoplasm with melanocytic differentiation and immunohistochemical positivity for S100, SOX10, Melan-A, and HMB45. Despite thrombectomy with iliac vein stenting, percutaneous nephrostomy tube placement, arterial embolization, and multidisciplinary oncologic management, the patient experienced progressive metastatic disease and ultimately transitioned to hospice care.

This case highlights the aggressive nature of anorectal melanoma and illustrates how advanced pelvic malignancy may produce complex multisystem complications requiring multidisciplinary management.

## Introduction

Anorectal melanoma is a rare malignancy accounting for approximately 1% of all melanomas and less than 2% of anorectal malignancies [1,2]. In contrast to cutaneous melanoma, mucosal melanomas arise from melanocytes within the mucosal epithelium and display distinct biological behavior, including aggressive local invasion and early metastatic spread [3].

Patients frequently present with nonspecific symptoms such as rectal bleeding, anorectal pain, tenesmus, or altered bowel habits. These symptoms often resemble benign anorectal disorders, including hemorrhoids, fissures, or polyps, contributing to delayed diagnosis and advanced disease at presentation [1,4]. Because early symptoms are subtle and frequently misattributed, prompt digital rectal examination, endoscopic evaluation, and low-threshold biopsy of suspicious anorectal lesions remain important practical strategies for earlier recognition [3,5].

Histopathologic examination with immunohistochemical analysis plays a central role in establishing the diagnosis. Markers such as S100 and SOX10 are highly sensitive for melanoma, while Melan-A and HMB45 provide additional specificity for melanocytic differentiation [5]. Staging of anorectal melanoma remains challenging because no universally adopted anorectal melanoma-specific American Joint Committee on Cancer (AJCC) staging system exists. In practice, many studies continue to apply the Ballantyne clinical staging system, in which stage I indicates localized disease, stage II indicates regional nodal disease, and stage III indicates distant metastases [6,7].

This report describes a patient with Ballantyne stage III anorectal melanoma complicated by massive pelvic tumor burden, hepatic metastases, malignant venous compression, and obstructive uropathy, illustrating the severe local and systemic complications that can arise in advanced pelvic malignancy.

## Case presentation

A 54-year-old female with a history of obesity presented to the emergency department with acute-onset generalized abdominal pain, malaise, urinary incontinence, and rectal bleeding. Symptoms began approximately two days before presentation. On arrival, she was hemodynamically stable but in acute distress with a pale conjunctiva.

Initial laboratory evaluation revealed leukocytosis, microcytic anemia, hyponatremia, hyperkalemia, and acute kidney injury (Table 1). Given the concern for gastrointestinal bleeding, gastroenterology was consulted, and a colonoscopy was performed. Endoscopic evaluation identified a friable rectal mass approximately 1 cm from the anal verge. No optical or endoscopic image of the lesion was available for inclusion in the medical record. Initial biopsy demonstrated poorly differentiated malignant cells, but it was insufficient to establish a definitive diagnosis.

**Table 1:** Pertinent laboratory findings BUN, blood urea nitrogen

Laboratory Test	Result	Units	Normal Reference Range	Clinical Note/Interpretation
Creatinine	5.6	mg/dL	0.52-1.04	Markedly elevated, consistent with severe acute kidney injury
BUN	69	mg/dL	7.0-17.0	Elevated, supportive of renal dysfunction
Sodium	123	mmol/L	137-145	Hyponatremia
Potassium	5.8	mmol/L	3.4-5.0	Hyperkalemia
Hemoglobin	7.5	g/dL	12.0-16.0	Severe anemia, likely multifactorial with tumor-related bleeding contribution
Hematocrit	23.3	%	37.0-47.0	Reduced
Mean corpuscular volume	79	fL	81-99	Microcytosis
Platelet count	173	µL	130-450x10^3^	Within normal limits
White blood cell count	21	µL	5.0-11.0x10^3^	Leukocytosis

The patient subsequently underwent transanal excision of the lesion. Histopathologic examination revealed a poorly differentiated malignant neoplasm with focal positivity for S100. Molecular testing identified NF1 and PIK3CA mutations, while gene fusions typical of clear cell sarcoma were absent, raising suspicion for melanoma.

Definitive diagnosis was established after a robotic abdominoperineal resection. Surgical pathology demonstrated a high-grade malignant neoplasm with melanocytic differentiation involving the rectum and invading the posterior vaginal wall. Immunohistochemical staining showed strong positivity for S100, SOX10, Melan-A, and HMB45, confirming rectal melanoma.

**Figure 1:**
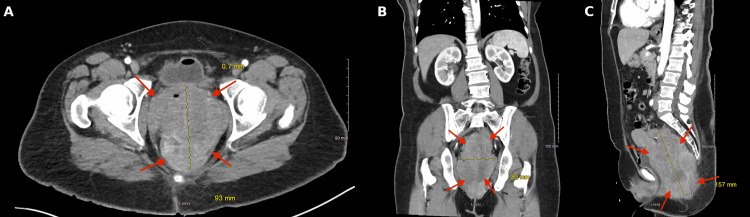
Contrast-enhanced CT of the abdomen and pelvis in the (A) axial, (B) coronal, and (C) sagittal views Contrast-enhanced CT demonstrates a large soft-tissue mass within the posterior pelvis. Arrows indicate the pelvic mass measuring approximately 93×83×157 mm occupying the rectouterine space. The mass displaces the urinary bladder and exerts a substantial pelvic mass effect, contributing to compression of adjacent vascular and urinary structures. CT, computed tomography

**Figure 2:**
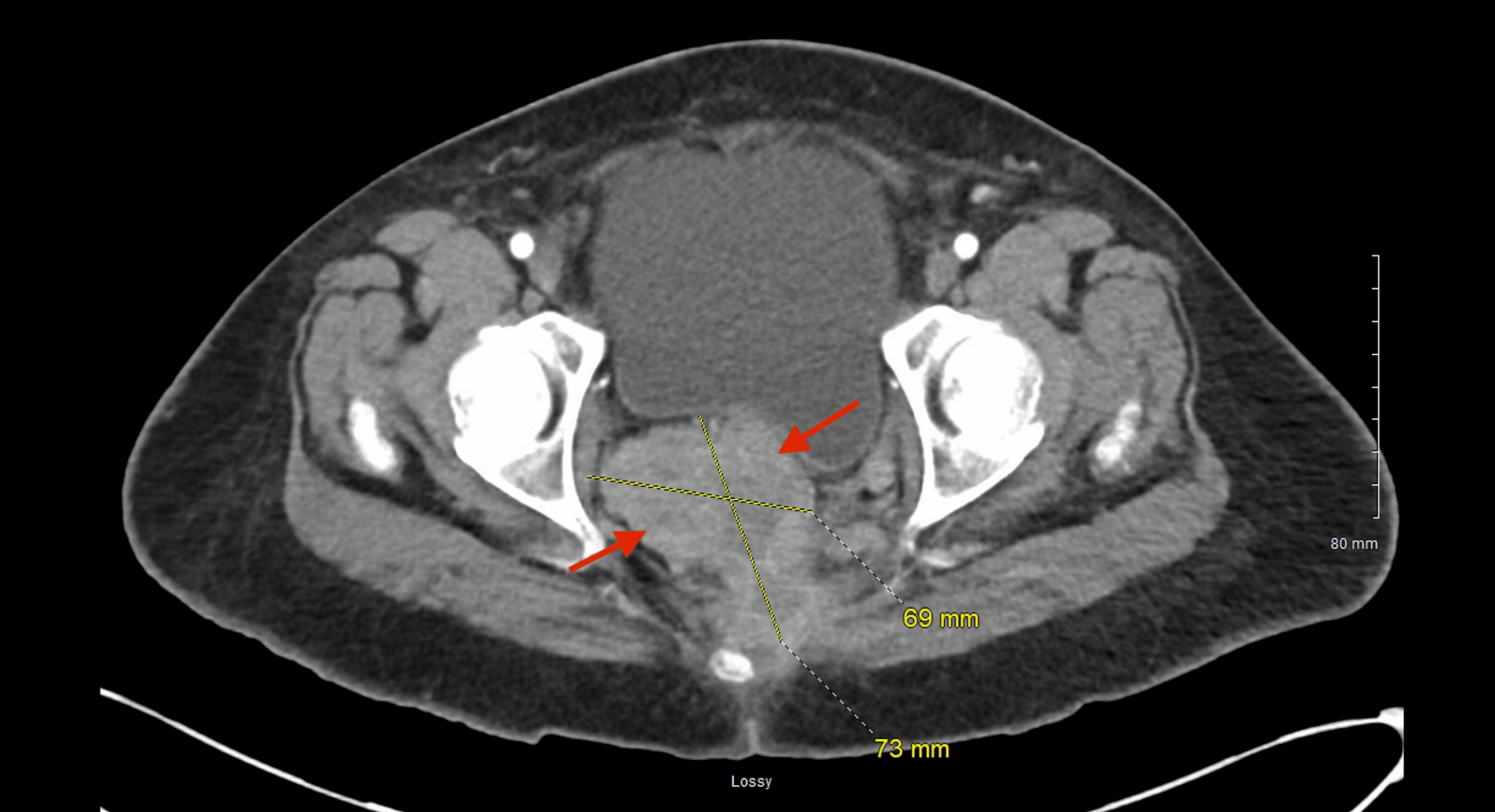
Contrast-enhanced CT of the abdomen and pelvis Contrast-enhanced CT demonstrates an enhancing rectal mass. Arrows indicate the rectal tumor measuring approximately 6.9×7.3 cm extending toward the anal and inguinal regions, with surrounding inflammatory change. This component contributes to local anorectal invasion and pelvic soft tissue distortion. CT, computed tomography

**Figure 3:**
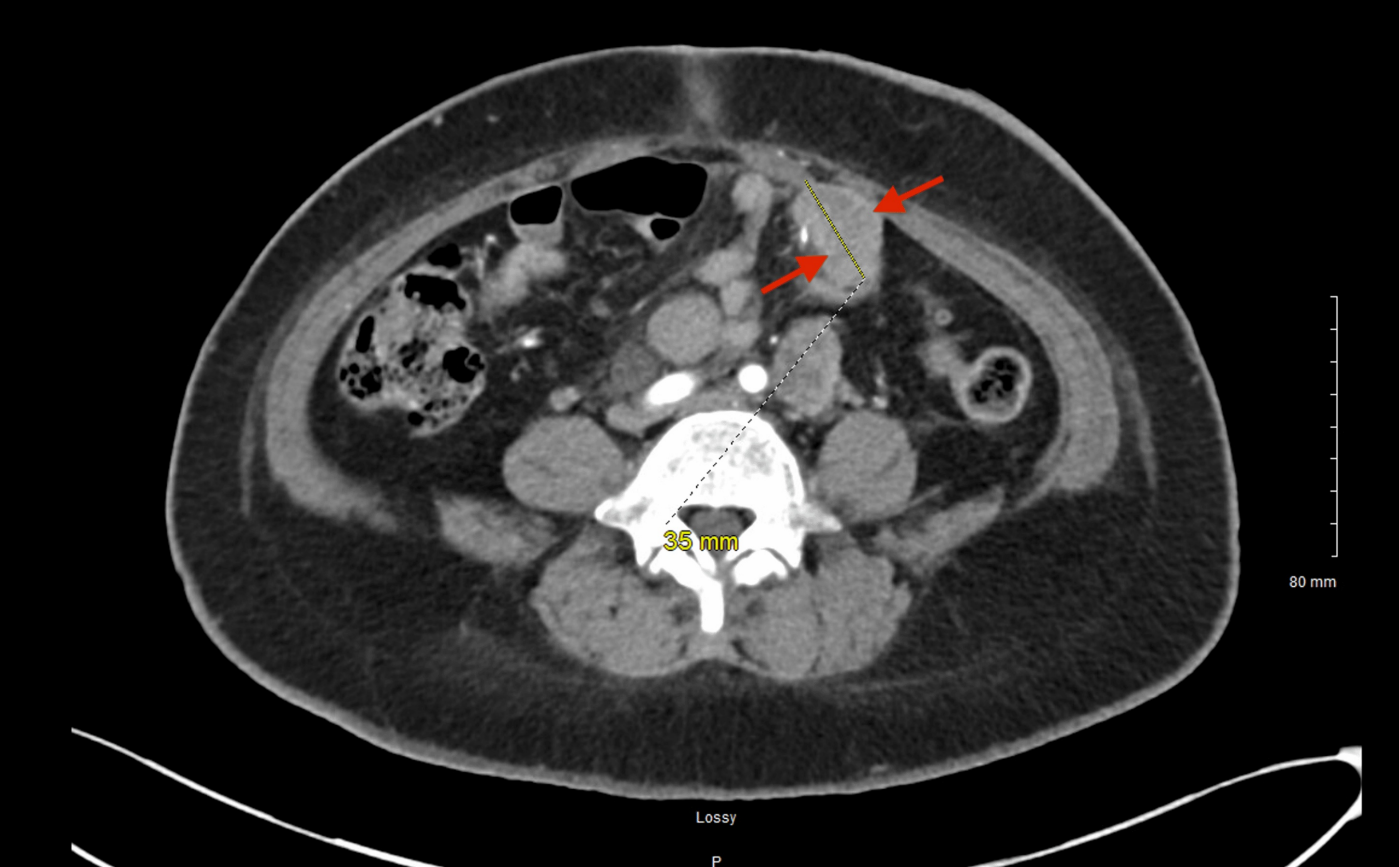
Contrast-enhanced CT of the abdomen and pelvis Contrast-enhanced CT demonstrates a focal enhancing lesion adjacent to the patient’s left lower quadrant colostomy. Arrows indicate a 3.5-cm enhancing lesion concerning for locally invasive metastatic disease. CT, computed tomography

Contrast-enhanced computed tomography (CT) demonstrated extensive pelvic tumor burden. A large posterior pelvic mass measuring approximately 93×83×157 mm occupied the rectouterine space and displaced adjacent pelvic structures, including the urinary bladder (Figure 1). An additional enhancing rectal mass measuring approximately 6.9×7.3 cm extended toward the anal and inguinal regions (Figure 2). A 3.5-cm enhancing lesion adjacent to the left lower quadrant colostomy was also identified and was suspicious for locally invasive metastatic disease (Figure 3).

**Figure 4:**
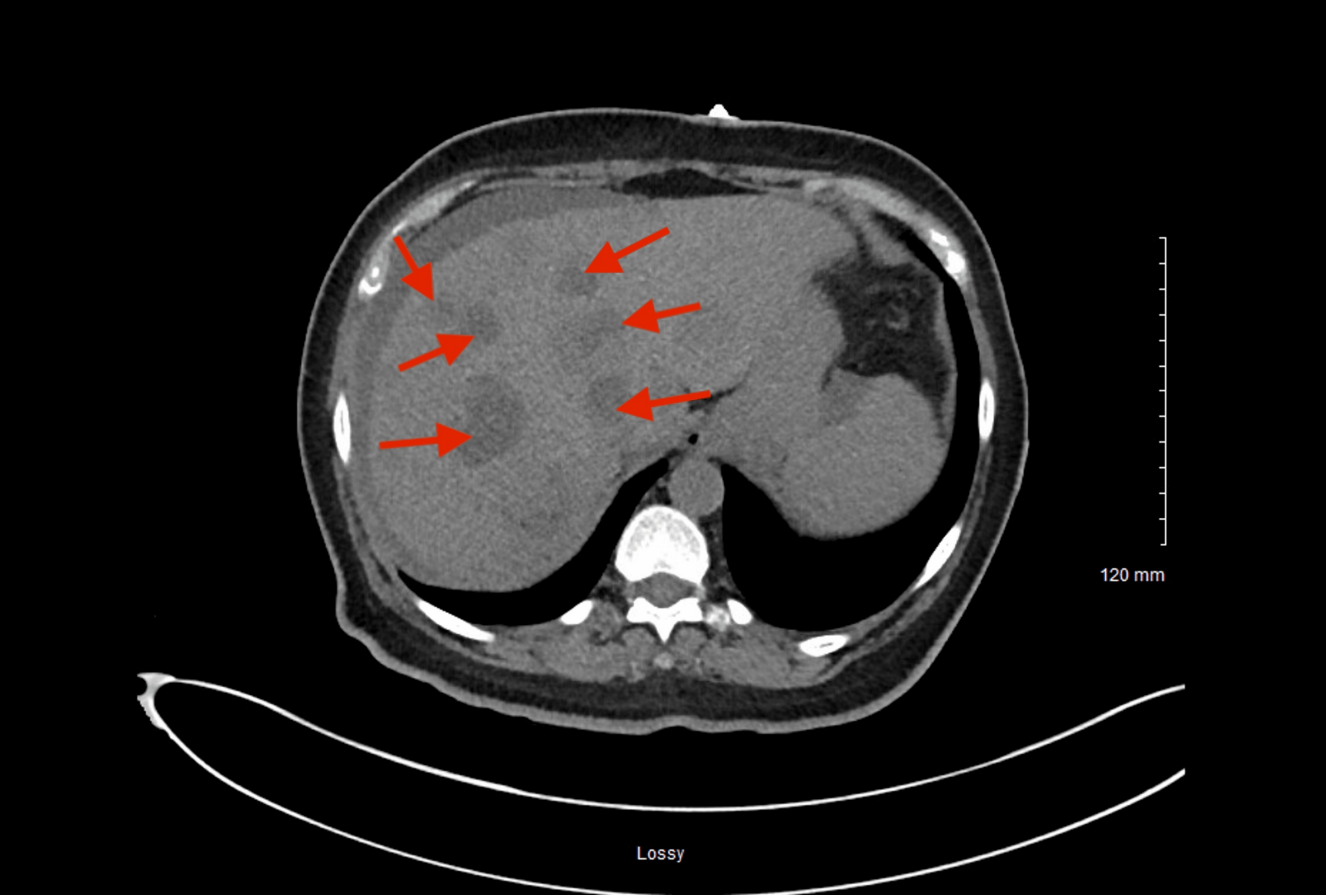
Contrast-enhanced CT of the abdomen and pelvis Contrast-enhanced CT demonstrates multiple hepatic lesions. Arrows indicate multiple hepatic metastases consistent with metastatic melanoma, establishing distant metastatic disease. CT, computed tomography

CT imaging further demonstrated multiple hepatic lesions consistent with metastatic disease without biliary ductal dilatation (Figure 4). Because distant liver metastases were present, the disease was classified as Ballantyne stage III anorectal melanoma [6,7].

Progressive tumor growth resulted in malignant compression of the left common iliac vein, leading to extensive deep venous thrombosis that required thrombectomy and iliac vein stent placement. Subsequent ureteral compression produced bilateral hydronephrosis requiring percutaneous nephrostomy tube placement. Persistent tumor-related bleeding required embolization of branches of the internal iliac arteries.

Despite aggressive multidisciplinary management, the patient experienced progressive metastatic disease and ultimately transitioned to hospice care (Table 2).

**Table 2:** Clinical timeline CT, computed tomography

Timepoint	Clinical Event
Initial presentation	Rectal bleeding prompting colonoscopy and discovery of rectal mass
Initial biopsy	Poorly differentiated malignant cells identified
Transanal excision	S100 positivity suggesting melanocytic differentiation
Molecular testing	NF1 and PIK3CA mutations detected
Definitive surgery	Abdominoperineal resection, confirming rectal melanoma
Staging imaging	CT demonstrating a massive pelvic tumor and hepatic metastases
Staging category	Ballantyne stage III disease due to distant hepatic metastases
Complications	Malignant iliac vein compression causing extensive deep venous thrombosis; bilateral hydronephrosis from ureteral compression
Interventions	Thrombectomy, iliac vein stent placement, nephrostomy tubes, arterial embolization
End-stage care	Transition to hospice because of progressive metastatic disease

## Discussion

Anorectal melanoma is among the most aggressive subtypes of mucosal melanoma and is associated with poor clinical outcomes. Reported five-year survival rates are often in the range of 10-20%, largely because diagnosis is delayed and metastatic spread occurs early [1-3]. Many patients present with regional or distant disease at the time of diagnosis, underscoring the importance of clinical suspicion when evaluating persistent rectal bleeding, anorectal pain, or an anorectal mass that does not behave like a benign lesion [1,3].

The diagnosis may be challenging because anorectal melanoma can appear poorly differentiated and may mimic carcinoma, lymphoma, or sarcoma on initial pathology. Immunohistochemical staining is therefore essential. S100 and SOX10 are highly sensitive markers of melanocytic lineage, whereas Melan-A and HMB45 provide additional specificity for melanocytic differentiation [5]. In this case, the sequential diagnostic process, from a nondiagnostic initial biopsy to transanal excision, molecular testing, and definitive surgical pathology, reflects the real-world difficulty of establishing the diagnosis in mucosal melanoma.

The extent of disease in this patient was particularly severe. Because hepatic metastases were present on staging imaging, this case meets criteria for Ballantyne stage III anorectal melanoma, the stage associated with distant metastatic disease [6,7]. Although alternative staging approaches have been explored, the Ballantyne system remains commonly used in the literature because a dedicated universally adopted AJCC staging framework for anorectal melanoma is lacking [6,7].

A major teaching point in this case is the impact of pelvic mass effect. Large pelvic tumors can compress adjacent veins, ureters, bladder, rectum, and pelvic soft tissues. In this patient, compression of the left common iliac vein likely contributed to venous stasis and extensive deep venous thrombosis, a recognized mechanism of cancer-associated thrombosis in patients with external tumor compression [8,9]. The tumor also produced obstructive uropathy with bilateral hydronephrosis, reflecting malignant ureteral obstruction from extrinsic compression, which can lead to post-renal acute kidney injury and frequently requires decompression with nephrostomy or stenting [10]. These complications demonstrate how anorectal melanoma may cause substantial morbidity not only through metastatic spread but also through destructive local pelvic invasion.

This case also raises the important question of earlier diagnosis. Earlier detection of anorectal melanoma remains difficult because presenting symptoms often overlap with hemorrhoids, fissures, rectal polyps, or nonspecific anorectal bleeding syndromes [1,3]. Practical opportunities for earlier recognition include thorough digital rectal examination, anoscopy or colonoscopy in patients with persistent or unexplained rectal bleeding, and prompt biopsy of atypical, pigmented, ulcerated, friable, or nonhealing anorectal lesions [3,5]. Earlier tissue diagnosis may not eliminate the aggressive biology of the disease, but it may reduce delays in staging and multidisciplinary treatment planning.

Management of advanced anorectal melanoma frequently requires coordination across surgical oncology, medical oncology, interventional radiology, urology, vascular medicine, and palliative care. In this case, venous thrombectomy with stenting, arterial embolization, and nephrostomy tube placement were all used as symptom-directed and complication-directed interventions. Even when a cure is not achievable, such measures may provide meaningful palliation and improve quality of life in advanced pelvic malignancy [8-10].

## Conclusions

Anorectal melanoma remains a rare but highly aggressive malignancy that is frequently diagnosed at an advanced stage. Because early symptoms often mimic benign anorectal conditions, clinicians should maintain a high index of suspicion when evaluating persistent rectal bleeding, anorectal pain, or a suspicious anorectal mass.

This case emphasizes the importance of histopathologic and immunohistochemical evaluation in establishing the diagnosis of mucosal melanoma. Cross-sectional imaging is also essential for assessing tumor burden, detecting metastatic disease, assigning stage, and identifying complications such as venous thrombosis and obstructive uropathy. Improved awareness of the aggressive behavior, staging implications, and mass-effect complications of anorectal melanoma may enhance earlier detection and guide multidisciplinary management in future cases.
